# Metabolomics and Proteomics Annotate Therapeutic Properties of Geniposide: Targeting and Regulating Multiple Perturbed Pathways

**DOI:** 10.1371/journal.pone.0071403

**Published:** 2013-08-15

**Authors:** Xijun Wang, Aihua Zhang, Guangli Yan, Wenjun Sun, Ying Han, Hui Sun

**Affiliations:** National TCM Key Lab of Serum Pharmacochemistry, Key Lab of Chinmedomics and Key Pharmacometabolomics Platform of Chinese Medicines, Heilongjiang University of Chinese Medicine, Harbin, China; Heidelberg University, Germany

## Abstract

Geniposide is an important constituent of *Gardenia jasminoides* Ellis, a famous Chinese medicinal plant, and has displayed bright prospects in prevention and therapy of hepatic injury (HI). Unfortunately, the working mechanisms of this compound are difficult to determine and thus remain unknown. To determine the mechanisms that underlie this compound, we conducted a systematic analysis of the therapeutic effects of geniposide using biochemistry, metabolomics and proteomics. Geniposide significantly intensified the therapeutic efficacy as indicated by our modern biochemical analysis. Metabolomics results indicate 9 ions in the positive mode as differentiating metabolites which were associated with perturbations in primary bile acid biosynthesis, butanoate metabolism, citrate cycle (TCA cycle), alanine, aspartate and glutamate metabolism. Of note, geniposide has potential pharmacological effect through regulating multiple perturbed pathways to normal state. In an attempt to address the benefits of geniposide based on the proteomics approaches, the protein-interacting networks were constructed to aid identifying the drug targets of geniposide. Six identified differential proteins appear to be involved in antioxidation and signal transduction, energy production, immunity, metabolism, chaperoning. These proteins were closely related in the protein-protein interaction network and the modulation of multiple vital physiological pathways. These data will help to understand the molecular therapeutic mechanisms of geniposide on hepatic damage rats. We also conclude that metabolomics and proteomics are powerful and versatile tools for both biomarker discovery and exploring the complex relationships between biological pathways and drug response, highlighting insights into drug discovery.

## Introduction

‘Omics’ technologies that are capable of measuring changes in large numbers of variables, often at a wide level, to build networks for analyzing drug action [1]. Combining omics and network analyses will enable the development of predictive models of therapeutic efficacy and for the identification of the target protein(s) for a compound with an unknown mechanism of action. Proteomic network analyses are emerging as a key tool in identifying potential drugs and allow us to develop an initial understanding of the context within which molecular-level drug-target interactions [2], [3]. As the newest of the “omics” sciences, metabolomics has been shown to have enormous potential when applied to subjects as diverse as drug mechanisms, disease processes, and drug discovery [4]–[6].

Knowledge about the protein targets of natural products is critical for understanding drug action [7]. To address drug effects in more physiological conditions, novel omics tools have recently been developed [8]. Liver injury has become a severe health problem worldwide. Despite considerable and continuous efforts, effective treatment strategies against this disease resulting in fewer side effects are still lacking. Oriental herbal medicines, widely used for treatment of various diseases, have recently attracted the interest of the modern scientific community as alternative therapies [9]. Zhizi (*Gardenia jasminoides* Ellis) is one of the most popular traditional medicinal plants for treatment of jaundice and liver disorders and has been used more than one thousand years (Chinese Pharmacopoeia Commission 2010). Interestingly, geniposide (Fig. S1), one of the biologically active ingredients main active constituents of gardenia fruit of Zhizi, has been proven effective in treating liver diseases, and shows hepato-protectivity and contributes directly to the therapeutic effect [10]–[12]. Such compound has distinct pharmacological effects, however, the precise mechanism of the action remains poorly understood.

Identification of the molecular targets of natural products is an important aspect of current drug discovery, as knowledge of the molecular targets will greatly aid drug development. The identification of their targets is required for a comprehensive understanding of their pharmacological role and for unraveling their mechanism of action [13]. Omics approaches combined with other biochemical methods can reconstruct regulatory networks, signaling cascades and metabolic pathways upon drug treatment [14], [15]. Herein we use the treatment of HI with geniposide as a working model, we conducted an investigation incorporating advanced metabolomic, proteomic technologies, and biochemical analyses. This is the first study that obtains a systematic view of dissection of mechanisms of geniposide as an effective treatment for liver injury.

## Results

### Geniposide Reduces Histologic Changes

**Figure 1:**
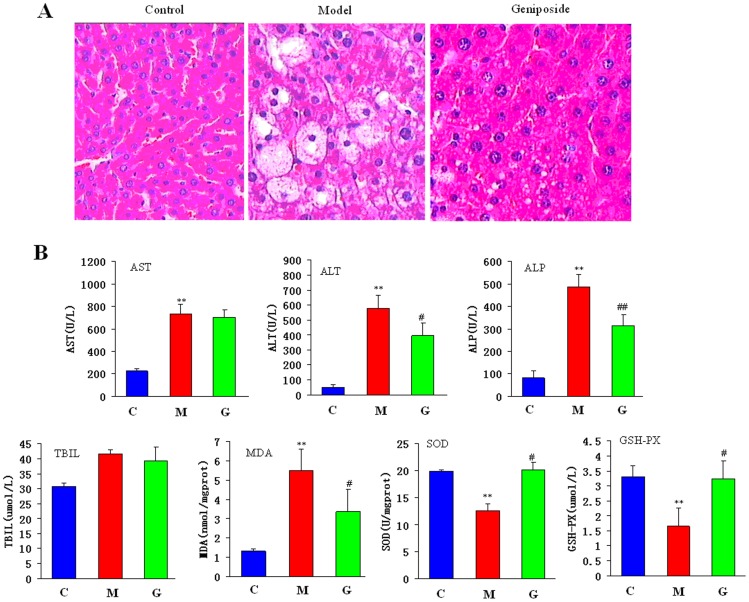
Representative pictures of liver histopathology after CCL_4_ with geniposide treatment in rats (original magnification: ×200); Therapeutic efficacy of geniposide as indicated by biochemical indicators. The values are expressed as mean± SD and were compared by ANOVA. ^*^P<0.05; ^**^P<0.01;^ #^P<0.05, ^##^P<0.01.

To confirm the protective effects of geniposide on liver tissue damage, histological analyses were performed in liver tissue that was obtained from HI rats and compared with tissue from control rats. Microscopic analyses of H&E-stained liver sections showed that geniposide significantly decreased hepatocyte necrosis, and fibrotic area, making them comparable to normal liver (Fig. 1A). The histopathological examination of liver sections that were stained with H&E revealed numerous apoptotic hepatocytes and the accumulation of massive necrosis with intralobular hemorrhage in the livers of HI rats. Minimal hepatocellular necrosis and inflammatory cell infiltration and mild portal inflammation were observed in rats that treated with geniposide. The degree of necrosis was clearly lower in the CCL_4_-treated rats that received geniposide as compared with animals that treated with the model. Furthermore, the H&E-stained sections indicate that hepatocyte size had changed in the model rats and that geniposide restored normal cell size. These results suggest that the geniposide prevents the destruction of liver tissue and intensifies therapeutic efficacy.

### Therapeutic Efficacy of Geniposide as Indicated by Biochemical Indicators

There was significant variation of the biochemical indicators such as ALT, AST, ALP, MDA, SOD and GSH-PX between the control and model groups (Fig. 1B). It indicated that the HI model successfully replicates the disease. The model group had higher ALT, AST, ALP, T-BIL, and MDA values but had lower levels of SOD and GSH-PX than the control animals. The indicators, SOD and GSH-PX results showed the recovery of the values by geniposide treatment, but values of AST and TBIL did not show any significant changes of values. Of note, the geniposide protocol decreased the levels of ALT, AST, ALP, T-BIL, and MDA but increased the levels of SOD and GSH-PX. Geniposide group was treated back to baseline levels of the control group, which demonstrates that this drug had a therapeutic effect in the rat HI model.

### Metabolomics Analysis

#### LC-MS analysis of metabolic profiling

**Figure 2:**
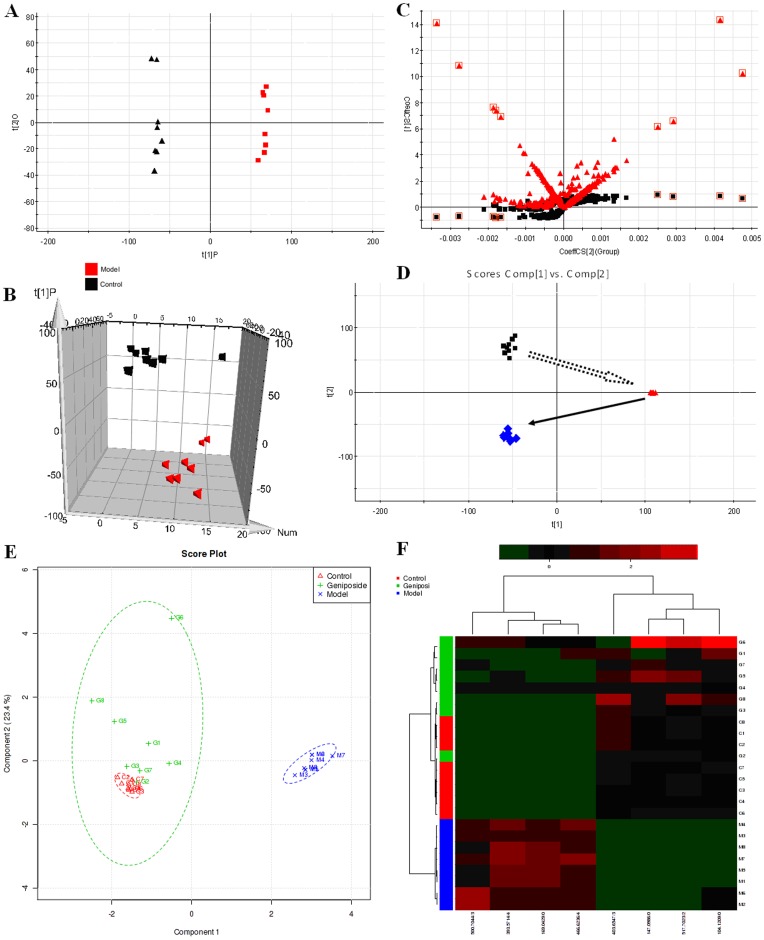
Metabolomic multivariate analysis. (A) PCA score plot for the control and HI groups. (black triangle, control group; red square, model group). (B) 3D-PCA plot for the control and HI groups; (C) A combination plot of S-plot and VIP values (EZinfo software 2.0). The red point graph is the VIP value plot, which represents the value of each metabolite. The farther from the origin, the higher the VIP value of the metabolites. (D) Trajectory analysis of PCA for the CCl_4_ treatment in positive mode. (E) Score plots for the acute livery injury after scoparone treatment in positive mode. (F): Heat map visualization for the urine samples. The heatmap was constructed based on the potential candidates of importance, which were extracted with PLS-DA analysis.

Using the optimal reversed-phase UPLC-MS conditions, all the data containing the retention time, peak intensity and exact mass were imported in the MasslynxTM software for multiple statistical analyses. Both PCA and PLS-DA often can be taken, because of their ability to cope with highly multivariate, noisy, collinear and possibly incomplete data. PCA is an unsupervised pattern recognition method initially used to discern the presence of inherent similarities in spectral profiles. Typically, the trajectory analysis of PCA in positive mode can really reflect the differences between the model and control groups, and showed metabolic profiles in the control and model group were separated clearly (Fig. 2A). Therefore, PCA can effectively demonstrate the differences between the treated and control groups. Fig. 2B demonstrates a clear separation between the treated and control groups. Nine ions showed significant difference in abundance between the control and treated animals and contributed to the observed separation were selected from the S-plot of PLS-DA (Fig. 2C). The farther away from the origin, the higher the VIP value of the ions was. Furthermore, PCA Score plot for the liver injury rats after geniposide treatment in positive model showed significant differences between samples (Fig. 2D), gradually back to the normal group. Combining the results of the PLS-DA analysis with PCA plots, the UPLC-MS analysis platform provided the retention time, precize molecular mass and MS/MS data for the structural identification of biomarkers. Finally, according to the variable importance in the projection, the ions (listed in Table S1).were identified based on the metabolite identification strategy [16]. The parallel analysis of the samples with MetaboAnalyst allows for the ability to verify that ions, which are identified through both ways (i.e. Score plots, Heatmaps), are highly significant, as depicted through two completely different algorithms. Analysis of the control, control and geniposide groups utilizing MetaboAnalyst’s score plot revealed differences among groups (Fig. 2E). The heatmap, commonly used for unsupervised clustering, were constructed based on the potential candidates of importance, which were extracted with PLS-DA analysis. Using our metabolomics platform, interestingly, the parallels heatmap visualization (Fig. 2F) using Ward’s method in computational systems analysis for the rats that treated with geniposide and model groups could be achieved the distinct segregation.

#### Changes of relative intensity of biomarkers

According to the protocol detailed above, 9 endogenous metabolites contributing to the separation of the model group and control group were detected in the urine samples. The ions identified by UPLC-MS are summarized in Table S1 with their corresponding retention time, m/z, ion mode, and related trends. The relative mean height intensity of different metabolites were graphed in Figure S2. Monitoring changes of these metabolites may predict the development of liver injury. Interestingly, the relative concentration of 9 endogenous metabolites was significantly affected by geniposide treatment. Additionally, compared with the alterations of liver injury-related metabolites, most of them were reset to a normal state after geniposide administration. Among them, 4 metabolites in the control values, and 2 in the model values are zero (Figure S2).

#### Biomarker network and metabolic pathway reconstruction

**Figure 3:**
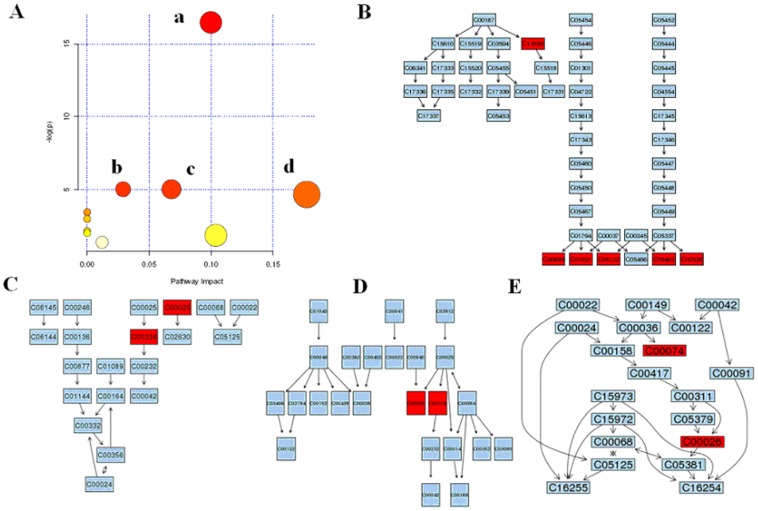
Summary of pathway analysis. **Identifying network pathway by MetPA software.** a, Primary bile acid biosynthesis; b, Butanoate metabolism; c, alanine, aspartate and glutamate metabolism; d, citrate cycle. Putative metabolic pathways of primary bile acid biosynthesis (B), butanoate metabolism (C), alanine, aspartate and glutamate metabolism (D), citrate cycle (E) metabolism were inferred in the CCl_4_ dosed rats from changes in the urine levels of intermediates during substance metabolism. Red denotes affected metabolites related to the pathway. The map was generated using the reference map by KEGG (http://www.genome.jp/kegg/).

With pattern recognition analysis of metabolites, a clear separation of model and control group was achieved, the geniposide group were located with control group. Metabolite profiling focuses on the analysis of a group of metabolites related to a specific metabolic pathway in biological states. To determine whether our observations of changes in the metabolites in the setting of liver injury in fact reflected coordinate changes in defined metabolic pathways, we used MetPA software to identify network pathway (Fig. 3A). This software was based on the high-quality KEGG metabolic pathways as the backend knowledgebase to help researchers identify the most relevant pathways involved in the conditions under study. The detailed construction of the metabolism pathways with higher score was shown in Fig. 3 and Table S2. MetPA revealed that top 4 pathways including primary bile acid biosynthesis (Fig. 3B), butanoate metabolism (Fig. 3C), citrate cycle (TCA cycle, Fig. 3D), alanine, aspartate and glutamate metabolism (Fig. 3E) *etc.* that changed specifically in the setting of HI. Results suggested that these target pathways showed the marked perturbations over the time-course of liver injury and could contribute to development of liver injury.

### Proteomics Analysis

#### 2-DE pattern and differential analysis of rat serum

Proteins from the control, model and geniposide groups were respectively solubilized in the IEF-compatible lysis buffer, and separated on IPGstrips and SDS-PAGE. Following staining with silver nitrate, the well-resolved and reproducible 2-D gel maps were obtained, which are displayed in Figure S3. Using PDQuest 2-DE gels analysis software, approximately 1000 well-stained, clearly-delineated protein spots were detected, and most spots were distributed in range from pI 3 to pI 10. Fifteen spots which showed remarkable change (Student’s t-test; p<0.05) between the model and control groups had been observed.

#### Proteins identified by MALDI-TOF-MS

Differentially expressed protein spots were excised from the 2-DE gel, and analyzed for protein identification by MALDI-TOF/TOF-MS. All PMFs were searched with Mascot software in SWISS-PROT database to identify the protein spots. The result had high confidence if the protein was ranked as the best hit with a significant score and high sequence coverage. Finally, we identified 15 proteins in those spots, which are shown in Table S3. A representative PMF map and database query result of protein zinc finger protein 407, alpha-1-antitrypsin and transthyretin were shown in Fig. S4. Relative Expression level of the differentially expressed proteins was shown in Figure S5. These proteins appear to be involved in antioxidation and signal transduction, energy production, immunity, metabolism, chaperoning. Hepatoprotective effects of geniposide on acute hepatic injury wistar rats was associated with regulated expression of six proteins (p<0.05) including zinc finger protein 407, haptoglobin, alpha-1-antitrypsin, glyceraldehyde-3-phosphate dehydrogenase, transthyretin and prothrombin.

#### Visualization and analysis of protein interaction networks

**Figure 4:**
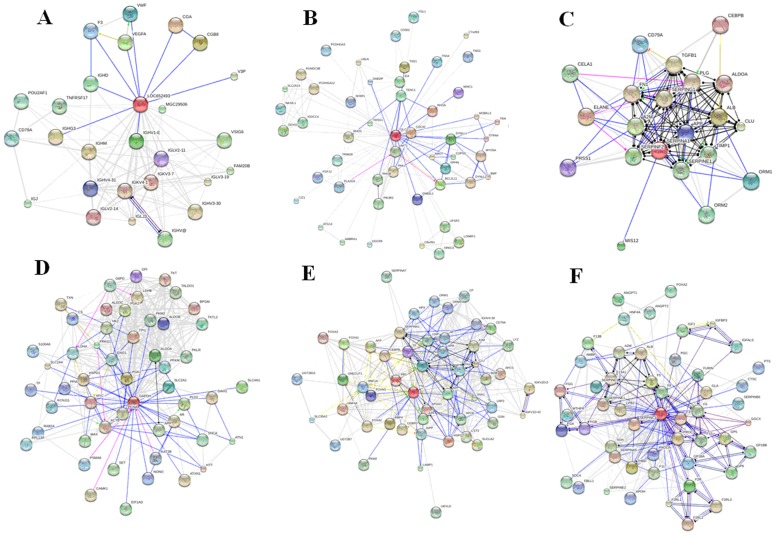
Interaction network generated for proteins identified. Modes of action are shown in different colors. Differentially expressed proteins are represented as pill-shaped nodes, while other proteins are shown as spheres. Nodes that are associated to each other are linked by an edge. Thicker lines represent stronger associations. Lines and, for directed edges, arrows of different colours stand for different edge types in the actions view: binding (blue), activation (green), inhibition (red), catalysis (magenta), same activity (cyan) and reaction (black). A: zinc finger protein 407; B: haptoglobin; C: alpha-1-antitrypsin; D: glyceraldehyde-3-phosphate dehydrogenase; E: transthyretin; F: prothrombin.

To facilitate access to the protein–protein interaction data, STRING integrates information about interactions from metabolic pathways, crystal structures, binding experiments and drug–target relationships. As seen in Fig. 4, association network of six differentially expressed proteins using STRING was constructed. To examine the protein functions and pathways of the differentially expressed proteins, the enriched GO terms were categorized for HI networks to identify the functional change. From all the GO categories covered by the differentially expressed proteins and their interactions, the identified proteins could be divided into several main groups according to major biological process categories: proteins related to antioxidation and signal transduction, protein involved in apoptosis, chaperone, energy generation, intermediary metabolism, regulation of cellular process, and molecular transport. These closely connected and coexpressed differentially expressed proteins in networks are regarded as the signatures of the underlying targets.

## Discussion

Tide of drug discovery has been turning back to Nature in the search for new drug candidates. Natural products have contributed to the development of important therapeutics to combat diseases over the past decades [17]. However, the targets and modes of action of these fascinating compounds are seldom known, hindering the drug development process. One example is geniposide, whose efficacy in treating HI has recently been well established [10], [11]. Because the means of action of geniposide on HI has not been sufficiently investigated, we used an ‘‘omics’’ platform and identified the key mechanisms that underlie the observed effects. Up to now, there have been no reports of the omics properties of geniposide in the relevant literature although this information is important to our further exploration of the geniposide as a drug candidate. To gain insight into the complex biochemical mechanisms of this effective HI therapy, we conducted an investigation that incorporated modern biochemical analyses, metabolomics and proteomics, analyzing the effects of the geniposide treatments on HI. We employed UPLC-HDMS, MALDI-TOF MS/MS, and 2-DE to systematically analyze the therapeutic properties of genipisode at the levels of the metabolome and proteome, thus exploring some of the key molecular mechanisms that underlie thes effects. It was demonstrated that based on UPLC-HDMS measurements of urine samples with statistically significant multivariate models could be constructed to distinguish between control and model subjects. The global metabolic profiling and subsequent multivariate analysis clearly distinguished HI rats from matched controls. As shown in Fig. 1A and B, PCA revealed an evident separation between the model and control samples. By using our metabolomics platform, 9 statistically important variables with VIP>6 were defined (Table S1). Interestingly, 9 distinct metabolites identified from these pathways, many are in various stages of progress at the HI. Further study of these metabolites may facilitate the development of non-invasive biomarkers and more efficient therapeutic strategies for HI. Furthermore, primary bile acid biosynthesis, butanoate metabolism, citrate cycle, alanine, aspartate and glutamate metabolism was the top function listed by Metaboanalyst tool. It is noteworthy that 9 metabolites together are important for the host response to HI through target metabolism pathways. A significant separation using Heatmap analysis was obtained for the difference between HI vs. controls as well as between genipisode vs. models. The HI rats were easily distinguished from control subjects by powerful Heatmap approach when using autoscaling methods. Based on the KEGG, a detailed construction of the primary bile acid biosynthesis, butanoate metabolism, citrate cycle, alanine, aspartate and glutamate metabolism pathways map with higher score is shown in Fig. 3. Our results indicated that these target pathways show the marked perturbations over the entire time-course of HI and could contribute to the development of HI. We have also built metabolomic features of geniposide protects against liver injury. Interestingly, geniposide exhibited hepatoprotective role of liver injury and kept animals in the normal situation, because there were no distinct clustering differences between control and geniposide group (Fig. 1D). In order to more clearly characterize treatment effects of geniposide, the intensity level of 9 markers in the different groups was analyzed (Figure S2) and, geniposide may regulate metabolism of these markers to efficiently used for liver injury disorder. Urinary excretion of these metabolites was also shown to have high specificity and sensitivity as markers. It indicated that these metabolites may be the biomarkers which were related to the action mechanism of geniposide.

Development and use of protein regulatory networks to mechanistically interpret this data is an important development in molecular biology, usually captured under the banner of systems biology. Several networks have recently been constructed to help drug discovery [18]–[22]. Additionally, predicting interactions between drugs and target proteins can help decipher the underlying biological mechanisms [23]. Therefore, in this study, to identify the possible target proteins of geniposide, 2-DE-based comparative proteomics was performed and proteins altered in expressional level after geniposide treatment were identified by MALDI-TOF MS/MS. Hepatoprotective effects of geniposide on acute HI wistar rats was associated with regulated expression of six proteins including zinc finger protein 407, haptoglobin, alpha-1-antitrypsin, glyceraldehyde-3-phosphate dehydrogenase, transthyretin and prothrombin. The change in the levels of these proteins caused by geniposide treatment appear to be involved in antioxidation and signal transduction, energy production, immunity, metabolism and chaperoning. As seen in Fig. S4, association network of differentially expressed proteins in the geniposide-treated group was constructed using STRING. Our study has identified robust biomarkers that are related to HI, with a special focus on the changes in the relative expression levels of target proteins after treatment. It is important to note that based on our results, geniposide targets not only immunity and metabolism but also targets key regulatory pathways that are used in transport, signal transduction, cell growth and proliferation, thereby helping to restore the normal function of the liver.

Modern drug discovery is time-consuming and expensive, involving coordinated multidisciplinary research in multiple stages, each requiring intensive and specialized resources. The rapid advancement of “omics” approaches have provided a vast array of significant information in life science, data relevant to drug discovery are not easily identified and recruited for application to pharmaceutical research [24]–[26]. Understanding of the action mechanism of geniposide will be helpful to the study and the use of likely promising natural protector [27]. Herein, we illustrate how omics can be utilized to explore the mechanisms of action of geniposide which affect different ‘key pathway’. It is the first demonstration of omic approach to delineate metabolic changes in liver liver injury after dosing geniposide treatment. Geniposide could have hepatoprotective effect and keep animals in the normal situation via multiple pathways including primary bile acid biosynthesis, and TCA cycle pathways *etc*. The potential target identification problem can be solved effectively by our method, for extracting differentially regulated subnetworks from protein-protein interaction networks based on data from global quantification technologies, which may become an effective strategy for the underlying mechanism of disease. Six proteins whose expressions were significantly changed under geniposide treatment were identified and might be considered as possible direct targets of geniposide. We conclude that omics is a powerful and versatile tool for both biomarker discovery and for exploring the complex relationships between biological pathways and drug response. The new methodology reported herein is anticipated to make an important contribution for elucidating the underlying biological mechanism of action at omics level toward drug discovery research in this area.

## Materials and Methods

### Chemicals and Reagents

Acetonitrile (HPLC grade) was purchased from Dikma Technology Inc. (Dima Company, USA). Deionized water was purified by theMilli-Q system (Millipore, Bedford, MA, USA). Leucine enkephalin and CCl4 were purchased from Sigma-Aldrich (MO, USA). CCl_4_ was supplied from Chemicals Factory (Beijing, P. R. China). Olive oil was supplied by Kerry Oils & Grains Trade Co., Ltd. (Shenzhen, China). Geniposide (purify 99%) were purchased from Sichuan Provincial Institute for Food and Drug Control (Sichuan, P. R. China). Ultra-pure reagents for polyacrylamide gel preparation were obtained from Bio-Rad. 18-cm immobilized pH gradient (IPG) strips, IPG buffer and dry strip cover fluid were purchased from Amersham Pharmacia Biotech (Uppsala, Sweden). Dithiothreitol (DTT), PMSF, iodoacetamide, urea, thiourea, α-cyano-4-hydroxycinnaic acid (CCA), and TPCK-Trypsin were purchased from Sigma Chemical (St. Louis, MO, USA). 3-[(3-cholamidopropyl) dimethylammonio]-propanesulfonate (CHAPS), glycine, ammonium persulphate (APS), TEMED, and trifluoroacetic acid were obtained from Amresco (Solon, OH, USA). The other reagents were purchased from Bio-Rad.

### Animal Handling and Sample Preparation

Male Wistar rats (weighting 240±20 g) were supplied by GLP Center of Heilongjiang University of Chinese Medicine (Harbin, China). The room temperature was regulated at 25±1°C with 40±5% humidity. A 12-h light/dark cycle was set, free access to standard diet and water. The animals were allowed to acclimatize for 7 days prior to dosing and putted in the metabolism cages during the urine collection periods specified below. After acclimatization, animals were randomly divided into 3 groups with 8 rats in each: the control; model; and geniposide groups. The rats in the control group were administrated with 0.9% saline in the whole procedure for 5 consecutive days. Rats were orally administrated with 20% CCl4 (1 ml/kg body weight) olive oil solution at forth day (6∶00 p.m.) to induce liver injury model for 5 consecutive days, and until day 8. Simultaneously, geniposide group was administrated with CCl4 and 0.7 mg/m drug treatment for 5 consecutive days. The rats were administrated an oral accurate volume of 1 mL/100 g of each animal. Urine was collected daily (at 6∶00 a.m.) from metabolism cages at ambient temperature throughout the whole procedure and centrifuged at 13,000 rpm at 4°C for 5 min, and the supernatants were stored frozen at −20°C until metabolomic analysis. Blood was collected from the hepatic portal vein, kept them on ice for 1 h and serum was separated via centrifugation at 4,500 rpm for 5 min at 4°C, flash frozen in liquid nitrogen and stored at −80°C until use. The experimental procedures were approved by the Animal Care and Ethics Committee at Heilongjiang University of Chinese Medicine (approval number: HUCM2010-116). All efforts were made to ameliorate suffering of animals.

### Biochemical Analysis and Histology Assay

We quantified the levels of serum ALT, AST, ALP, T-BIL, MDA, SOD, and GSH-PX content using assay kits according to the manufacturer’s instructions. The rat livers were removed immediately after plasma collection and stored at −70°C until analysis. The livers were fixed in 4% neutral buffered formaldehyde at 4°C and embedded in paraffin. The liver tissue was stained with H&E for histopathological analysis.

### Metabolic Profiling

#### Chromatography

UPLC/ESI-Q-TOF/MS was used for the global analysis of urine samples. Chromatographic analysis was performed in a Waters ACQUITY UPLC system controlled with Masslynx (V4.1, Waters Corporation, Milford, USA). An aliquot of 6 µL of sample solution was injected onto an ACQUITY UPLC BEH C_18_ column (50 mm×2.1 mm i.d.,1.7 µm, Waters Corp, Milford, MA, USA) at 35°C and the ﬂow rate was 0.5 mL/min. The optimal mobile phase consisted of a linear gradient system of (A) 0.1% formic acid in acetonitrile and (B) 0.1% formic acid in water, 0–8.2 min, 90–30% A; 8.2–8.5 min, 30–1%A; 8.5–10.0 min, 1%A; 10–10.5 min, 1–99%A; 10.5–12.5 min, 99%A. In addition, the QC sample was used to optimize the condition of UPLC-Q-TOF/MS, as it contained most information of whole urine samples. Whenever one sample injection was finished, a needle wash cycle was done to remove the remnants and prepare for the next sample. In addition, the eluent was transferred to the mass spectrometer directly, that is, without a split.

#### Mass spectrometry

For the urine UPLC-HDMS (Waters Corporation, Milford, USA) analysis, the optimal conditions for positive ion mode were as follows: capillary voltage of 2,500 V, desolvation temperature of 350°C, sample cone voltage of 35 V, extraction cone voltage of 3 V, microchannel plate voltage of 1,600 V, collision energy of 4 eV, source temperature of 110°C, cone gas flow of 50 L/h and desolvation gas flow of 600 L/h. A lockmass of leucine enkephalin at a concentration of 200 pg/mL in acetonitrile (0.1% formic acid): H_2_O (0.1% formic acid) (50∶50, v/v) for positive ion mode ([M+H]^+^ = 556.2771) was employed via a lock-spray interface. The data were collected in centroid mode, the lock spray frequency was set at 5 s and the lock mass data were averaged over 10 scans. The mass spectrometry full-scan data were acquired in the positive ion mode from 100 to 1000 Da with a 0.1-s scan time.

#### Multivariate data analysis

MS data were generated recorded using MassLynx V4.1 (Waters Corporation, Milford, USA), MarkerLynx Application Manager (Waters Corporation, Milford, USA) was used for peak detection, and EZinfo software (which is included in MarkerLynx Application Manager and can be applied directly) was used for the “Unsupervised” and principal component analysis (PCA), “Supervised” partial least squares-discriminant analysis (PLS-DA) and orthogonal projection to latent structures (OPLS) analysis. All the variables were mean-centered and Pareto-scaled prior to PCA and PLS-DA. In the PLS-DA modeling, the samples from the different groups were sorted into different classes using score plots, and endogenous metabolites that contribute to the classification were identified in loading plots, which showed the importance of each variable to the classification. Each score plot has a loading plot associated with it, which makes it possible to identify the spectral regions (metabolites) that are responsible for the observed sample clustering.

#### Biomarkers identification and reconstruction of metabolic pathway

Potential markers of interest were extracted from S-plot (EZinfo software 2.0) constructed following analysis with PLS-DA, and markers were chosen based on their contribution to the variation and correlation within the data set. Statistical analysis was conducted using MANOVA via direct comparisons of biochemical parameters and metabolite concentrations (i.e., comparisons between model and control subjects with respect to these characteristics). The MassFragment™ application manager (Waters corp., Milford, USA) was used to facilitate the MS fragment ion analysis process by way of chemically intelligent peak-matching algorithms. With regard to the identification of biomarkers, the ion spectrum of potential biomarkers was matched with the structure message of metabolites acquired from available biochemical databases, such as HMDB, http://www.hmdb.ca/; METLIN, http://metlin.scripps.edu/; MassBank, http://www.massbank.jp/; and Lipidmaps, http://www.lipidmaps.org/. The reconstruction, interaction and pathway analysis of potential biomarkers was performed with MetPA online (http://metpa.metabolomics.ca) based above database source to identify the metabolic pathways. MetPA uses the high-quality KEGG metabolic pathways as the backend knowledgebase.

### Proteomics Analysis

#### Two-dimensional gel electrophoresis

The plasma obtained was disrupted in a homogenization buffer containing 30 mM Tris, 7 M urea, 2 M thiourea, 4% CHAPS, and 40 mM DTT, after which the lysates were centrifuged at 12,000 rpm for 60 min at 4°C. The supernatant was precipitated using the TCA sediment method to recover the total proteins, and the interfering components were removed using the 2-D Clean-Up Kit (GE Healthcare, Piscataway, NJ). The supernatant was removed, divided into several aliquots and stored at −70°C until use. The protein concentration of each sample was measured using the Bradford method. A total of 350 mg of protein was loaded onto an 18-cm linear IPG strip (pH 3–10, Amersham Biosciences, Piscataway, NJ) for first-dimension isoelectric focusing (IEF). The strips were placed into a Protean IEF cell (Bio-Rad) and rehydrated at 50 V for 12 h, after which the proteins were separated based on their pI according to the following protocol: 250 V with linear climb for 60 min, 1,000 V with rapid climb for 60 min, 10,000 V with linear climb for 5 h, and 10,000 V with rapid climb until a level of 60,000 V-h was reached. After IEF, the IPG strips were equilibrated for 15 min in a buffer containing 50 mM Tris-HCl, pH 8.8, 30% glycerol, 7 M urea, 2% SDS, and 1% DTT; they were then further treated in a similar buffer (containing 4% iodoacetamide) for 15 min and directly applied onto 12% homogeneous SDS-PAGE gels for 6 h electrophoresis using a Protean II xi cell system (Bio-Rad). Each experiment was performed in triplicate.

#### Image analysis

The gels were then silver-stained using Bio-Rad Silver Stain Plus kit reagents (Bio-Rad) according to the manufacturer’s instructions. The silver-stained gels were scanned using a GS-800 densitometer (Bio-Rad) and then analyzed using PDQuest software (Bio-Rad). Labscan Software (Applied Biosystems, Foster City, CA) was used for image analysis that included background abstraction, spot intensity calibration, spot detection, and matching. The intensity of each spot was quantified by calculating the spot volume after the gel image had been normalized. Each sample was processed in triplicate to ensure reproducibility, and the Student’s t-test was used to evaluate the average change in protein abundance corresponding to each target spot across the gels. Protein spots with more than a 3-fold significant change in density (p<0.05) were considered to be differentially expressed and were selected for further identification using MALDI-TOF MS/MS.

#### Protein identification using MALDI-TOF-MS and database searching

Protein spots of interest with differential expression levels were excised from the 2-D gels with an EXQuest spot cutter (Bio-Rad), washed 3 times in distilled water, destained with 15 mM potassium ferricyanide in 50 mM sodium thiosulfate for 2 min at room temperature, dehydrated in acetonitrile and finally dried in a centrifugal vaporizer. After a 30-min incubation, the gels were digested for more than 12 h at 37°C. The peptides were then extracted twice using 0.1% TFA (trifluoroacetic acid) in 50% CAN dried under the protection of N_2_. For MALDI-TOF/TOF MS, the peptides were mixed with 0.5 µL MALDI matrix (5 mg/ml α-cyano-4-hydroxycinnamic acid diluted in 0.1%TFA and 50% ACN) and spotted onto the MALDI sample plates. The MS measurements were performed on a Proteomics Analyzer with delayed ion extraction (Applied Biosystems). The parameters of MALDI-TOF were set up as follows: accelerating voltage 20 kV; grid voltage 64.5%; delay 100 ns; the number of laser shots 50 and the acquisition mass range 900 Da-3,500 Da. MS/MS accuracy was calibrated against the MS/MS fragments of m/z, which is one of the peaks that is generated in the myoglobin peptide mass fingerprint (PMF). The parameters for peak matching were as follows: the minimum signal-to-noise ratio was 20, the mass tolerance was 0.2 Da, the minimum peak to match reference masses was 4, and the maximum outlier error was 50 ppm. Bioinformatics databases were searched for protein identification based on tryptic fragment sizes. PMF were searched in the SWISS-PROT database (http://www.expasy.org/) using Mascot software (http://www.matrixscience.com). The protein score is −10*Log(P), where P is the probability that the observed match is a random event. The missed cleavage sites allowed were up to 1, and scores that were higher than 61 were considered significant.

#### Bioinformatics analysis of proteomic data

Identified proteins were further analysed using STRING, chosen as the source for protein-protein interactions, to statistically determine the functions and pathways most strongly associated with the protein list. Prior to upload and analysis using STRING, the mean ratio of each quantified protein in a group was calculated and the fold change between the groups calculated. The PANTHER cassification system and KEGG (www.genome.jp/kegg/) pathway database were used to further examine the significantly expressed molecules.

### Statistical Analyses

All statistical analyses were performed using the Student’s t-test. Differences with a P-value of 0.05 or less were considered significant. Assays were performed in triplicate, and the results are expressed as mean±SD.

## Supporting Information

Figure S1 **Chemical structures of geniposide.**
(TIF)

Figure S2 **Relative expression level of the differentially expressed metabolites.**
(TIF)

Figure S3 **A representative two-dimensional electrophoresis (2-DE) representative proteomic maps of control (A), model (B) and scoparone (C) group.**
(TIF)

Figure S4 **Peptide mass fingerprint spectrum of zinc finger protein 407, alpha-1-antitrypsin, transthyretin.** The spot was in-gel digested with trypsin. After desalted, the peptide mixture was analyzed by MALDI-TOF-MS. The x-axis represents the mass-to-charge ratio (m/z), and the y-axis represents the relative abundance. All protein identifications are provided in Table S2. Down: the probability-based Mowse scores obtained using the Mascot search engine. Among predicted proteins with differential Mowse scores shown as multiple bars on the x-axis, only proteins with Mowse scores greater than 61 were considered significant, which were 305, 303 and 397 (p<0.05) for ainc finger protein 407, alpha-1-antitrypsin, transthyretin, respectively.
(TIF)

Figure S5 **Relative expression level of the differentially expressed proteins obtained by MALDI-TOF/MS.**
(TIF)

Table S1 **Identification of urinary biomarkers in HI cases.**
(DOC)

Table S2 **Result from Pathway Analysis.**
(DOC)

Table S3 **Results of identifications of differentially expressed proteins using MALDI-TOF/TOF MS.**
(DOC)
