# Supplementary figures and images for: Metabolomics and Proteomics Annotate Therapeutic Properties of Geniposide: Targeting and Regulating Multiple Perturbed Pathways
Source: PLoS One. 2013 Aug 15;8(8):e71403. doi: 10.1371/journal.pone.0071403 (PMC3744542; doi:10.1371/journal.pone.0071403)

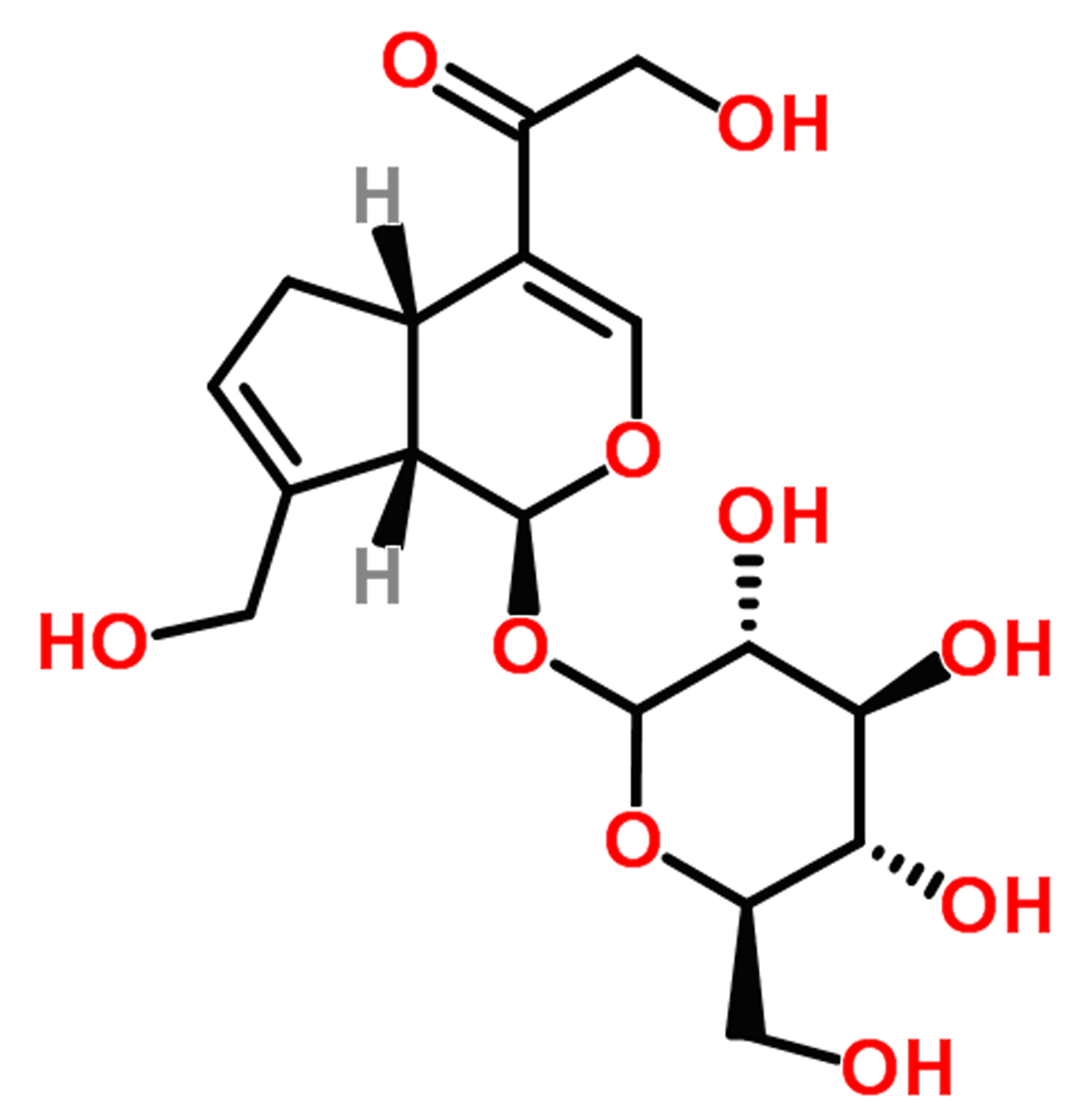

Supplement: Figure S1 — Chemical structures of geniposide. (TIF) [file pone.0071403.s001.tif]

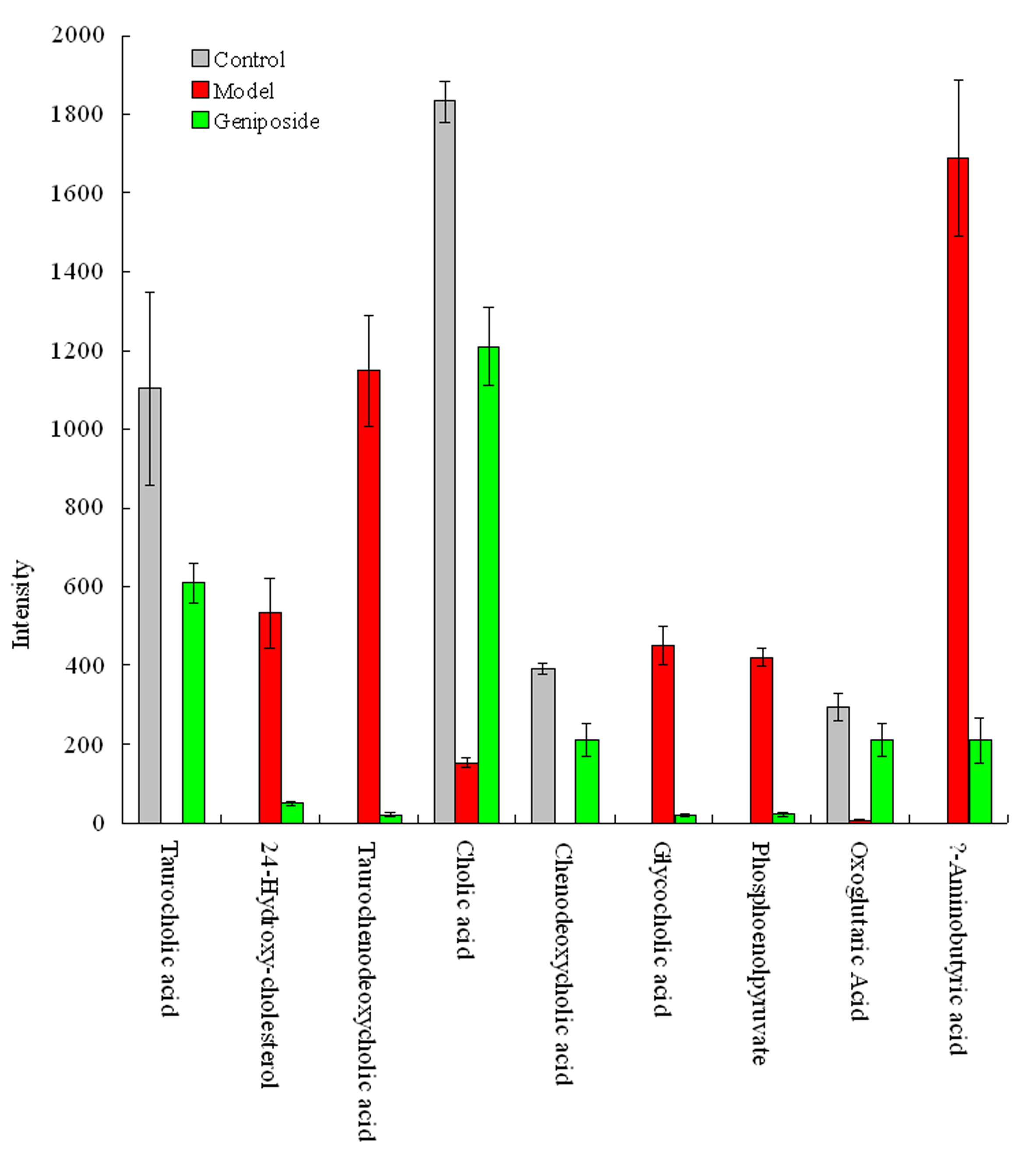

Supplement: Figure S2 — Relative expression level of the differentially expressed metabolites. (TIF) [file pone.0071403.s002.tif]

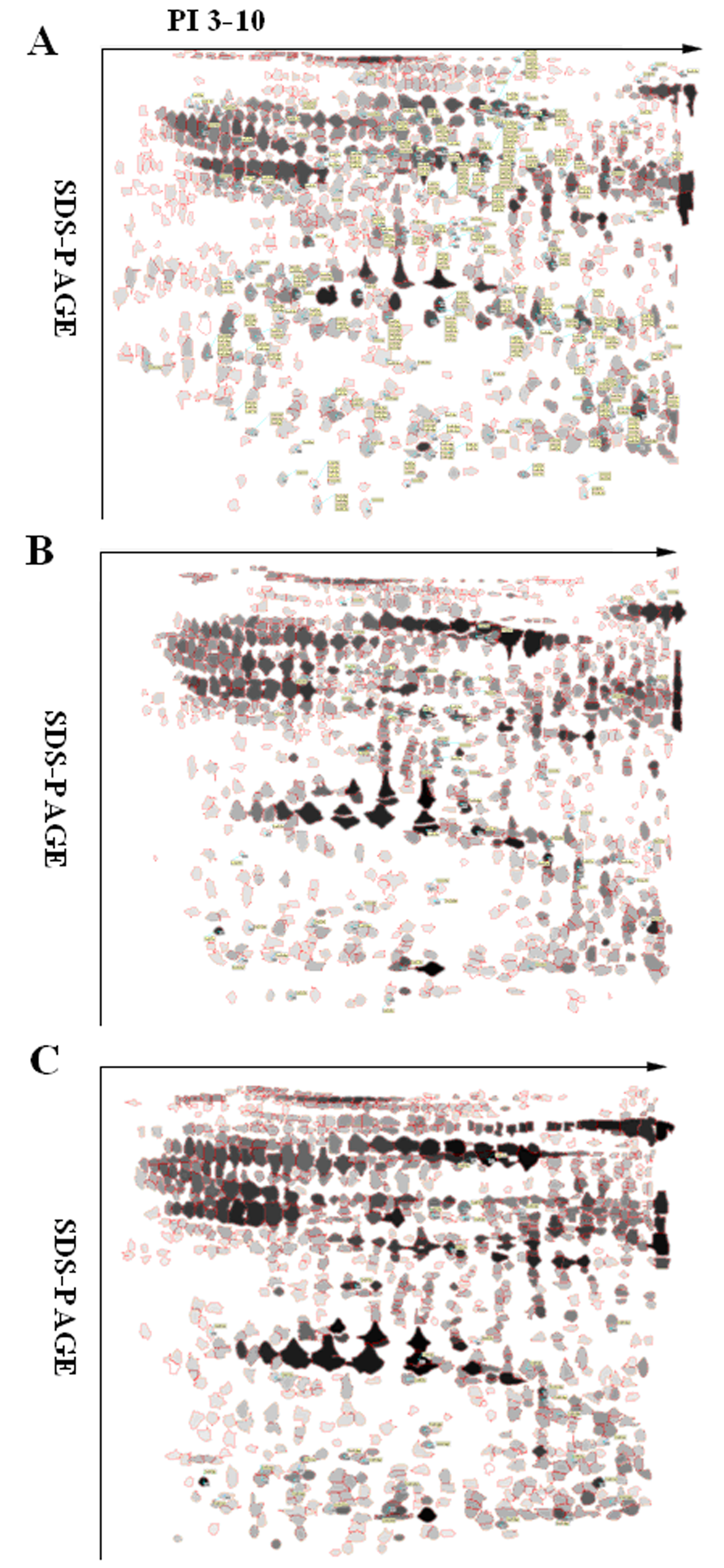

Supplement: Figure S3 — A representative two-dimensional electrophoresis (2-DE) representative proteomic maps of control (A), model (B) and scoparone (C) group. (TIF) [file pone.0071403.s003.tif]

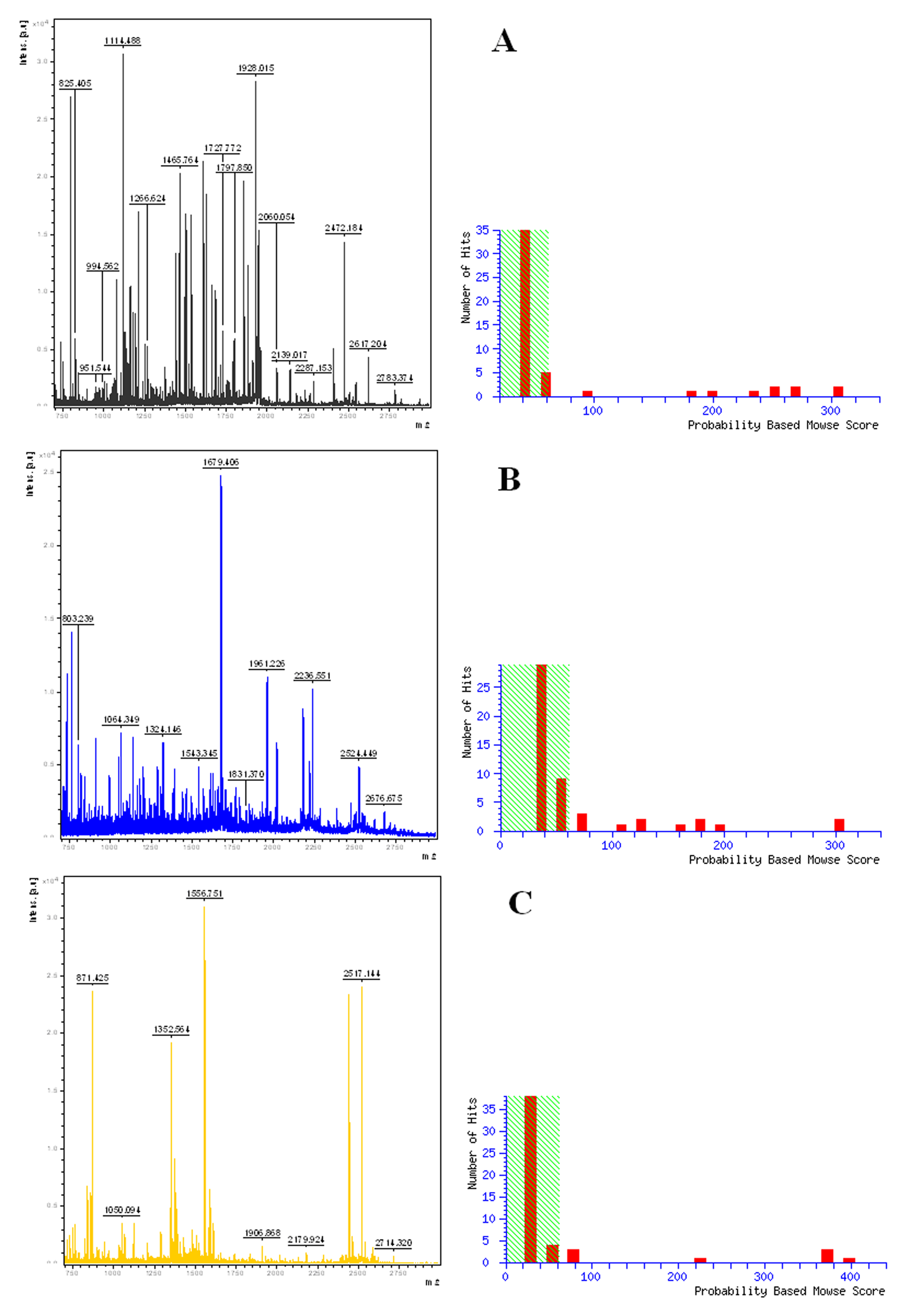

Supplement: Figure S4 — Peptide mass fingerprint spectrum of zinc finger protein 407, alpha-1-antitrypsin, transthyretin. The spot was in-gel digested with trypsin. After desalted, the peptide mixture was analyzed by MALDI-TOF-MS. The x-axis represents the mass-to-charge ratio (m/z), and the y-axis represents the relative abundance. All protein identifications are provided in Table S2. Down: the probability-based Mowse scores obtained using the Mascot search engine. Among predicted proteins with differential Mowse scores shown as multiple bars on the x-axis, only proteins with Mowse scores greater than 61 were considered significant, which were 305, 303 and 397 (p<0.05) for ainc finger protein 407, alpha-1-antitrypsin, transthyretin, respectively. (TIF) [file pone.0071403.s004.tif]

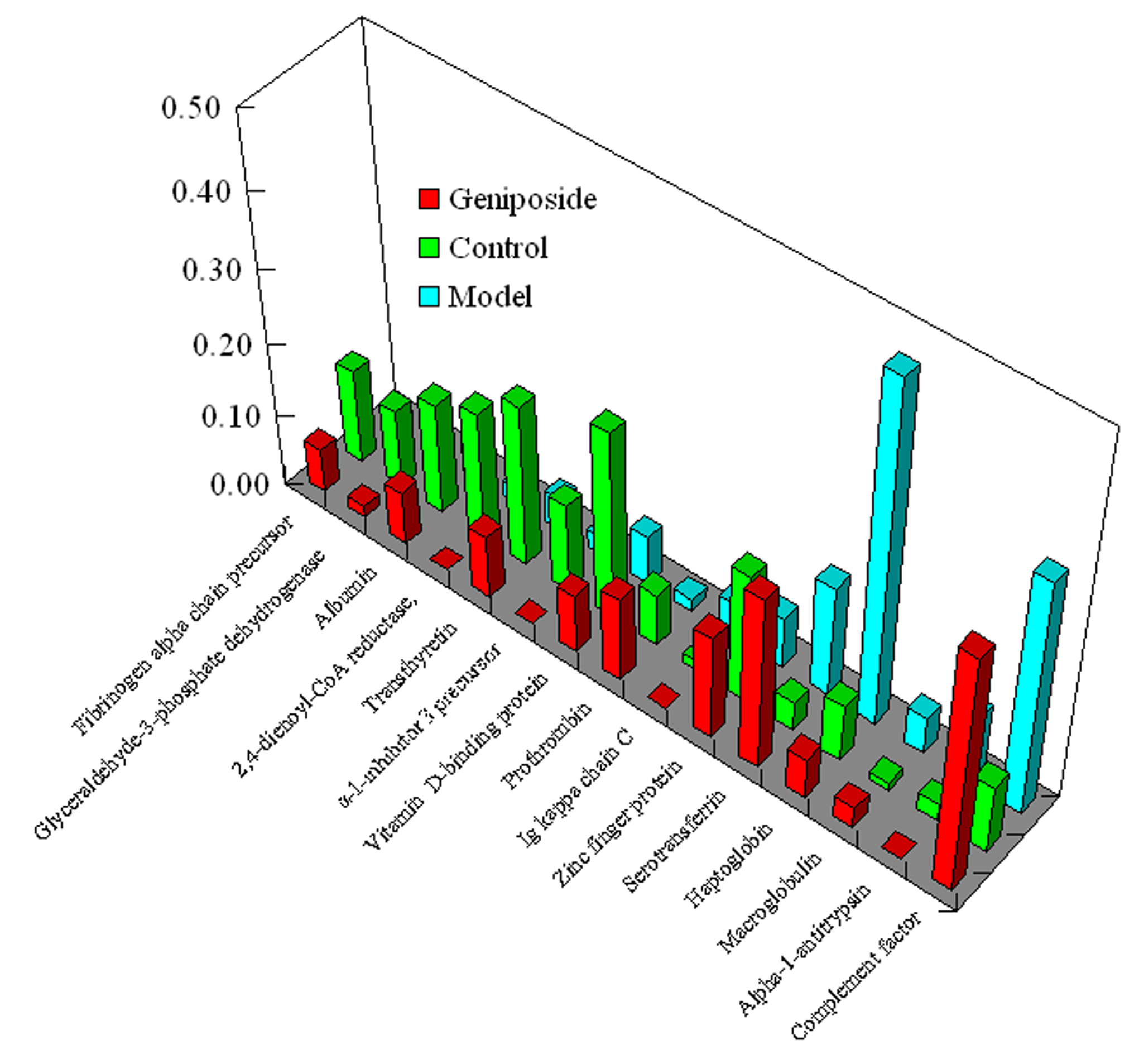

Supplement: Figure S5 — Relative expression level of the differentially expressed proteins obtained by MALDI-TOF/MS. (TIF) [file pone.0071403.s005.tif]
