# Supplementary material for: Metabolomics and Proteomics Annotate Therapeutic Properties of Geniposide: Targeting and Regulating Multiple Perturbed Pathways
Source: PLoS One. 2013 Aug 15;8(8):e71403. doi: 10.1371/journal.pone.0071403 (PMC3744542; doi:10.1371/journal.pone.0071403)
Supplement: Table S1 — Identification of urinary biomarkers in HI cases. (DOC) [file pone.0071403.s006.doc]

**Table S1.** Identification of urinary biomarkers in HI cases.

| **No.** | **VIP** | **Rt** | **[M+H]+** | **Mass** | **Formula** | **Metabolites** | **Trend** | **P-value** |
| --- | --- | --- | --- | --- | --- | --- | --- | --- |
| 1 | 14.3244 | 2.9 | 517.7023 | 515.7030 | [C_26_H_45_NO_7_S](http://www.chemspider.com/Molecular-Formula/C26H45NO7S) | Taurocholic acid | ↓ | 0.03 |
| 2 | 14.0655 | 3.48 | 403.6547 | 402.6529 | [C_27_H_46_O_2_](http://www.chemspider.com/Molecular-Formula/C27H46O2) | 2,4-Hydroxy-cholesterol | ↑ | 0.01 |
| 3 | 10.8291 | 3.48 | 500.7044 | 499.7036 | [C_26_H_45_NO_6_S](http://www.chemspider.com/Molecular-Formula/C26H45NO6S) | Taurochenodeoxycholic acid | ↑ | 0.02 |
| 4 | 10.2183 | 3.49 | 409.5726 | 408.5714 | [C_24_H_40_O_5_](http://www.chemspider.com/Molecular-Formula/C24H40O5) | cholic acid | ↓ | 0.05 |
| 5 | 7.38963 | 4.64 | 393.5714 | 392.5720 | [C_24_H_40_O_4_](http://www.chemspider.com/Molecular-Formula/C24H40O4) | chenodeoxycholic acid | ↑ | 0.00 |
| 6 | 7.64012 | 4.15 | 466.6236 | 465.6227 | [C_26_H_43_NO_6_](http://www.chemspider.com/Molecular-Formula/C26H43NO6) | Glycocholic acid | ↓ | 0.00 |
| 7 | 6.91141 | 0.71 | 169.0429 | 168.0420 | [C_3_H_5_O_6_P](http://www.chemspider.com/Molecular-Formula/C3H5O6P) | Phosphoenolpyruvate | ↓ | 0.00 |
| 8 | 6.57841 | 0.63 | 147.0986 | 146.0980 | [C_5_H_6_O_5_](http://www.chemspider.com/Molecular-Formula/C5H6O5) | Oxoglutaric Acid | ↑ | 0.02 |
| 9 | 6.11655 | 0.65 | 104.1209 | 103.1200 | [C_4_H_9_NO_2_](http://www.chemspider.com/Molecular-Formula/C4H9NO2) | γ-Aminobutyric acid | ↓ | 0.01 |
