# Supplementary material for: Metabolomics and Proteomics Annotate Therapeutic Properties of Geniposide: Targeting and Regulating Multiple Perturbed Pathways
Source: PLoS One. 2013 Aug 15;8(8):e71403. doi: 10.1371/journal.pone.0071403 (PMC3744542; doi:10.1371/journal.pone.0071403)
Supplement: Table S2 — Result from Pathway Analysis. (DOC) [file pone.0071403.s007.doc]

**Table S2**. Result from Pathway Analysis

| **Pathway** | **Total** | **Expected** | **Hits** | **Raw p** | **-log(p)** | **Impact** |
| --- | --- | --- | --- | --- | --- | --- |
| Primary bile acid biosynthesis | 46 | 0.29529 | 6 | 7.00E-08 | 16.475 | 0.09985 |
| Butanoate metabolism | 20 | 0.12839 | 2 | 0.006558 | 5.0271 | 0.02899 |
| Citrate cycle (TCA cycle) | 20 | 0.12839 | 2 | 0.006558 | 5.0271 | 0.06799 |
| Alanine, aspartate and glutamate metabolism | 24 | 0.15407 | 2 | 0.0094 | 4.6671 | 0.17721 |
| D-Glutamine and D-glutamate metabolism | 5 | 0.032097 | 1 | 0.031732 | 3.4504 | 0 |
| Taurine and hypotaurine metabolism | 8 | 0.051355 | 1 | 0.050339 | 2.989 | 0 |
| beta-Alanine metabolism | 19 | 0.12197 | 1 | 0.11588 | 2.1552 | 0 |
| Pyruvate metabolism | 22 | 0.14123 | 1 | 0.13304 | 2.0171 | 0 |
| Glycolysis or Gluconeogenesis | 26 | 0.1669 | 1 | 0.15546 | 1.8614 | 0.10389 |
| Arginine and proline metabolism | 44 | 0.28245 | 1 | 0.2501 | 1.3859 | 0.01198 |
