# Supplementary material for: Metabolomics and Proteomics Annotate Therapeutic Properties of Geniposide: Targeting and Regulating Multiple Perturbed Pathways
Source: PLoS One. 2013 Aug 15;8(8):e71403. doi: 10.1371/journal.pone.0071403 (PMC3744542; doi:10.1371/journal.pone.0071403)
Supplement: Table S3 — Results of identifications of differentially expressed proteins using MALDI-TOF/TOF MS. (DOC) [file pone.0071403.s008.doc]

**Table S3**. Results of identifications of differentially expressed proteins using MALDI-TOF/TOF MS.

| **Database ID no.** | **Identified protein name** | **MASCOT score** | **PI** | **MW** |
| --- | --- | --- | --- | --- |
| gi|125144 | Ig kappa chain C | 185 | 5.02 | 26051 |
| gi|55628 | Zinc finger protein | 489 | 6.49 | 34886 |
| gi|61556986 | Serotransferrin | 275 | 5.87 | 53532 |
| gi|33086640 | Haptoglobin | 318 | 5.71 | 53590 |
| gi|12084772 | Macroglobulin | 250 | 5.83 | 54878 |
| gi|203063 | Alpha-1-antitrypsin | 101 | 5.07 | 61662 |
| gi|46237594 | Complement factor | 97 | 5.96 | 77040 |
| gi|71824 | Fibrinogen alpha chain precursor | 131 | 6.43 | 38765 |
| gi|510196 | Glyceraldehyde-3-phosphate dehydrogenase | 140 | 7.56 | 31613 |
| gi|158138568 | Albumin | 208 | 6.75 | 34348 |
| gi|17105350 | 2,4-dienoyl-CoA reductase, | 103 | 4.71 | 36531 |
| gi|136467 | Transthyretin | 397 | 6.29 | 42190 |
| gi|83816939 | α-1-inhibitor 3 precursor | 303 | 5.84119 | 60934 |
| gi|203941 | Vitamin D-binding protein | 126 | 5.44 | 62398 |
| gi|135809 | Prothrombin | 156 | 5.26 | 91978 |

Note: Relative molecular weight (MW); Isoelectric point (PI)
